# Influence of Commonly Used Endodontic Irrigants on the Setting Time and Metal Composition of Various Base Endodontic Sealers

**DOI:** 10.3390/polym14010027

**Published:** 2021-12-22

**Authors:** Jerry Jose, Kavalipurapu Venkata Teja, Manish Ranjan, Roshan Noor Mohamed, Mohammad Khursheed Alam, Deepti Shrivastava, Valentino Natoli, Anil Kumar Nagarajappa, Krishnamachari Janani, Kumar Chandan Srivastava

**Affiliations:** 1 Department of Conservative Dentistry & Endodontics, Saveetha Dental College & Hospitals, Saveetha Institute of Medical &Technical Sciences, Saveetha University, Chennai 600077, India; jerryjosekavungal@gmail.com (J.J.); venkatatejak.sdc@saveetha.com (K.V.T.); 2 Department of Pediatric Dentistry, Faculty of Dentistry, Taif University, P.O. Box 11099, Taif 21944, Saudi Arabia; roshan.noor@tudent.edu.sa; 3 Orthodontics, Department of Preventive Dentistry, College of Dentistry, Jouf University, Sakaka 72345, Saudi Arabia; mkalam@ju.edu.sa; 4 Periodontics, Department of Preventive Dentistry, College of Dentistry, Jouf University, Sakaka 72345, Saudi Arabia; 5 Department of Dentistry, School of Biomedical and Health Sciences, European University of Madrid, 28670 Madrid, Spain; 6 Private Dental Practice, 72015 Fasano, Italy; 7 Oral Medicine & Radiology, Department of Oral & Maxillofacial Surgery & Diagnostic Sciences, College of Dentistry, Jouf University, Sakaka 72345, Saudi Arabia; dr.anil.kumar@jodent.org (A.K.N.); drkcs.omr@gmail.com (K.C.S.); 8 Department of Conservative Dentistry and Endodontics, SRM Institute of Science and Technology, SRM Dental College, Chennai 600089, India; jananik6@srmist.edu.in

**Keywords:** endodontics, root canal irrigants, heavy metals, root canal filling materials

## Abstract

The present study aimed to evaluate if commonly used endodontic irrigants such as 3% sodium hypochlorite (NaOCl, Prime Dental, Thane, India), 2% chlorhexidine (CHX, Sigma-Aldrich Co., St. Louis, MO, USA), and 17% ethylenediaminetetraacetic acid (EDTA, Meta-Biomed Co. Ltd., Cheongju-si, South Korea) influenced the setting time and metal composition of different base endodontic sealers on exposure. AH Plus (Dentsply De Trey GmbH, Konstanz, Germany), Sealapex (SybronEndo, Orange, CA, USA), mineral trioxide aggregate (MTA) Fillapex (Angelus Soluções Odontológicas, Londrina, Brazil), and Tubli-Seal (Kerr Dental, Orange, CA, USA) were selected as the different base representatives of endodontic sealers. These sealers were exposed to 3% NaOCl, 2% CHX, and 17% EDTA, and the individual setting time of the sealers was analyzed. The samples were analyzed for heavy metal elements such as chromium (Cr), nickel (Ni), cobalt (Co), cadmium (Cd), arsenic (As), mercury (Hg), lead (Pb), and beryllium (Be) by using inductively coupled plasma mass spectrometry (ICP-MS) analysis. For statistical analysis, one-way ANOVA and post hoc Tukey’s tests were used. All selected sealers showed variation in setting time post-exposure to different irrigants. MTA Fillapex had the shortest mean setting time (215.7 min, post-exposure at 187.3 min) (*p* < 0.05). Mean setting time was also affected for AH Plus (479.6 min, post-exposure at 423.9 min) (*p* < 0.05) and Tubli-Seal (514.7 min, post-exposure at 465.2 min) (*p* < 0.05). Sealapex showed the maximum reduction of setting time (864.8 min, post-exposure at 673.4 min) (*p* < 0.05). All tested sealers showed heavy metals (Cr, Ni, Co, Cd, As, Hg, and Pb) in their composition, and the quantities were influenced by interaction with different irrigants. The heavy metal Be was not seen in any of the samples. Sealapex showed the longest setting time in comparison to other test sealers. Heavy metals were most present in Sealapex, followed by AH Plus, Tubli-Seal, and MTA Fillapex. MTA Fillapex was seen to have the shortest setting time, and heavy metal composition was least affected on interaction with different commonly used endodontic irrigants. Further, this study provides significant insight into the influence of different endodontic irrigants on interaction with different base endodontic sealers, which has not been reported previously, and future studies should emphasize endodontic irrigant-sealer interactions and their possible effects in the long run.

## 1. Introduction

Endodontic sealers are considered to be one of the fundamental requisites of endodontic therapy, with the primary function to fill voids and infiltrate areas of the root canal system such as lateral and accessory canals where the core obturating material fails to reach, resulting in a complete seal of the root canal complexities [1]. According to Dag Ørstavik [2], endodontic sealers by design come in direct contact with the instrumented dentinal surface, forming a seal on the tubular structures, depleting the source of nutrition, and preventing further ingress for microorganisms into the canal in the long run. With the paradigm shift of the constituents of endodontic sealers into different bases such as zinc oxide-eugenol (ZOE), resin, mineral trioxide aggregate (MTA), and calcium hydroxide [Ca(OH)_2_] endodontic sealers, significant progress has been demonstrated in endodontic practice, with each exhibiting its own merits and demerits [3,4]. However, some reports suggest that different base characteristics in the sealers can influence the cytotoxic and genotoxic levels of human tissues in the long run [5,6].

Currently, numerous base endodontic sealers are available, each marketed by different manufacturers. From a historical perspective, zinc oxide-based sealers are one of the longest-used endodontic sealers; initially introduced by Grossman in 1958,they were reported to exhibit significant advantages and were priorly used as the standard for many laboratory-based studies [7]. Based on this, Tubli-Seal (Kerr Dental, Orange, CA, USA) was introduced, which showed advantages such as less cytotoxic effect on extraradicular cells and better dissolution properties, compared to epoxy-based sealers [8,9]. In 1995, one of the representatives of epoxy resin-based sealers, AH Plus (Dentsply De Trey GmbH, Konstanz, Germany), was introduced as a two-component system and had properties such as adequate seal, wettability, and antimicrobial activity [10]. The usage of salicylate resin as one of the components in the endodontic sealer matrix is proven to influence the physicochemical and cytotoxicity properties of the sealer [11]. With the introduction of calcium hydroxide-based sealers with salicylate components such as Sealapex (SybronEndo, Orange, CA, USA), a significant increase in antibacterial properties was witnessed, mainly due to alkaline pH properties causing denaturation of bacterial proteins, making them less toxic to vital tissues. Additionally, they stimulate osteogenic and cementogenic properties of bone-forming cells in comparison to resin-based sealer [12,13]. The recent introduction of MTA-based sealers has produced significant advancements in the properties of endodontic sealers, such as better periapical healing properties and biocompatibility compared with various other endodontic materials [14]. MTA Fillapex (Angelus Soluções Odontológicas, Londrina, Brazil), though not a true representative of this class of sealers, which consists of both Portland cement and butyl ethylene glycol disalicylate with a high resin/MTA ratio, has been shown to exhibit lower viscosity, lower solubility, better bioactivity, and better osteoconductive properties, compared to other base endodontic sealers [15].

After an extensive search, it was seen that elemental analysis of sealer-based materials has been explored less in endodontic literature. The presence of heavy metals such as arsenic (As), cadmium (Cd), cobalt (Co), mercury (Hg), lead (Pb), chromium (Cr), nickel (Ni), and beryllium (Be), singularly or in combination, can influence the physicochemical properties of endodontic biomaterials to a certain extent [16]. In addition, they exhibit some level of carcinogenic effect on cells in the long run. For instance, it is seen that As can inhibit cellular functions and distort intracellular microstructures of human cells upon constant exposure. Due to this, recommendations have been implemented that the quantity of As present should be limited to 2 mg/kg (2 ppm) according to the International Standards Organization (ISO) 9917-1:2003 standard [17].

Sodium hypochlorite (NaOCl), chlorhexidine (CHX), and ethylenediaminetetraacetic acid (EDTA) are the most commonly used endodontic irrigants used in endodontics, primarily for their properties such as antimicrobial mechanism, smear layer removal, and tissue-dissolving action [18]. However, to our knowledge, no studies have reported whether endodontic irrigants could influence the setting properties of different base endodontic sealers. Concerning this, the present study aimed to evaluate if commonly used endodontic irrigants such as 3% NaOCl, 2% CHX, or 17% EDTA influenced the setting time and metal composition on exposure to different base endodontic sealers. The null hypothesis for the present study was that there was no influence on the setting time and composition of different base endodontic sealers upon exposure to commonly used endodontic irrigants.

## 2. Materials and Methods

### 2.1. Study Characteristics

This in-vitro, experimental study was approved by the ethics committee of Saveetha University (ethical approval code: IHEC/SDC/ENDO-1805/21/34).

### 2.2. Materials

The study was carried out under strict aseptic in-vitro conditions. The composition and the manufacturers of the different sealers used for the study are shown in Table 1. These sealers were divided into subgroups based on exposure to commonly used endodontic irrigants such as 3% NaOCl (Prime Dental, Thane, India), 2% CHX (Sigma-Aldrich Co., St. Louis, MO, USA), and 17% EDTA (Meta—Biomed Co. Ltd., Cheongju-si, South Korea). The total group was considered to be 16, with further division into 4 subgroups based on the interaction of endodontic sealers with different endodontic irrigants.

### 2.3. Sample Preparation

Freshly extracted mandibular premolars for orthodontic or periodontal reasons were selected for this study. Any teeth that exhibited caries, fractured restoration, immature root apices, and a high degree of curvature determined via radiographs were excluded. After extraction, the roots of teeth were curetted and placed in a 5% formalin solution (Fisher Scientific, Mumbai, India). Each tooth was then decoronated using a diamond disk, up to the level of the cementoenamel junction such that only 12 mm of the root was achieved. The canal anatomy was further verified using a cone beam computed tomography (CBCT)scan (CS9600, Carestream Health, Inc., Rochester, NY, USA) using the following parameters: voxel size of 0.075 mm, 120 kVp, and 4.0 mA, and FOV of the image was adjusted to 8 × 5 cm, with 15.0 s scan time.

Teeth that exhibited single root with patent canals with curvature less than 5° and round canals were selected for this study, following canal patency filling using ISO No. 10stainless-steel K File (Mani, Tochigi, Japan). The glide path preparation was carried out using the Proglider system (Dentsply Maillefer, Ballaigues, Switzerland) (250 RPM, 1.0 Ncm^2^), and sequential preparation of the canals was done using the ProTaper Gold system (Dentsply Maillefer, Ballaigues, Switzerland) (300 RPM, 3.0 Ncm^2^) such that all the specimens had an apical preparation standardized to size 25, 0.06 taper (Supplementary document Figure S1). The irrigation protocol was based on previously published evidence [19] for all the specimens and was carried out using a 30G side-vented single port needle (Neoendo, Gurugram, India) such that it was present 2 mm from the apical foramen. Intermittent irrigation during the instrumentation process was conducted using 5 mL of 3% NaOCl for a 3 min period and 2 mL of 17% EDTA for another 2 min period, followed by irrigation with 2 mL of distilled water for all the samples. Based on exposure of samples of different base endodontic sealers to different endodontic irrigants, the final irrigation protocol varied and was carried out as follows:

- Sealers not exposed to any endodontic irrigants: final irrigation was conducted with 2 mL of distilled water for a 1 min period;
- For 3% NaOCl Group: final irrigation was conducted with 2 mL of 3% NaOCl for a 1minperiod;
- For 2% CHX Group: final irrigation was conducted with 2 mL of 2% CHX for a 1 min period;
- For 17% EDTA Group: final irrigation was conducted with 2 mL of 17% EDTA for a 1 min period.

Postexposure, the canals were dried using 0.06 taper paper points (Diadent, Cheongju-si, South Korea).The sealers were mixed according to the manufacturer’s instructions on a sterile glass slab, followed by which the prepared canals were filled with respective sealers with the help of asize-25 lentulospiral (Dentsply Maillefer, Ballaigues, Switzerland) attached to a motorized handpiece (300 RPM, 1 Ncm^2^) for 10 min (Supplementary document Figure S1), allowing the remaining irrigants present in the dentinal surface to incorporate evenly into the sealers. The sealers were then filled to the prepared working length (12 mm) (Supplementary document Figure S2), and the verification of the fill of the sealer till the apex was done using a digital periapical radiograph. The specimens were then divided according to the different selected groups and subjected to setting time and metal element analysis.

### 2.4. Setting Time Analysis

**Figure 1:**
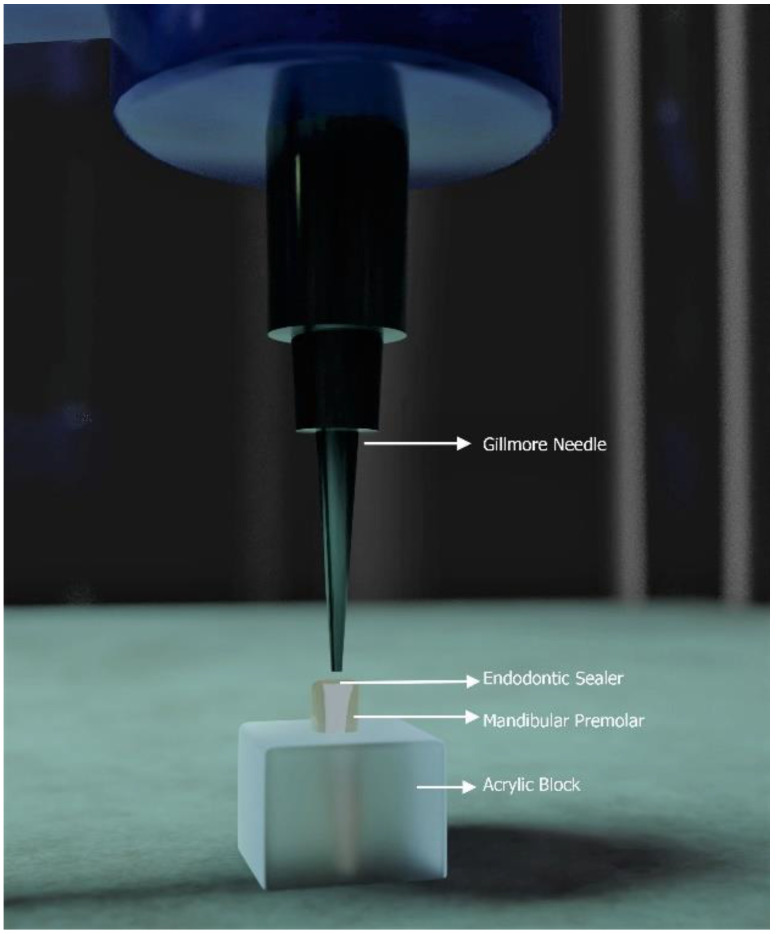
Schematic representation of the setting time analysis apparatus used for this study using a 100 g Gillmore needle set in an incubator.

The selected specimens with sealers were embedded in an acrylic block with an internal diameter of 10 mm and thickness of 2 mm, such that the decoronated coronal section was exposed to the operator (Figure 1). The setting time of each sealer was evaluated according to the ISO 6876:2012 standard [20]. The specimens were kept in an incubator at 37 °C and 95% humidity, and a visual qualitative determination by two specialists was done to evaluate the degree of the sealer. These specialists were blinded from the sealer they were examining. The sealer present on the decoronated tooth was first assessed with the naked eye, the fractured surface was viewed under a microscope, and the visible sealer was tested with a small handheld needle (27G needle). In case of disagreement among the two evaluators, the third evaluator was enquired to reach a consensus. A 100 g Gillmore needle with a 2 mm active tip was placed on the sample surface vertically. Indentations were repeated in a 2 min interval in 1 h followed by a 30 min interval after 1 h. The initial setting time was considered when the indentations of the needle were not seen on the sealer surface. Each sample was repeated five times, and the mean of the samples was taken as the final values. The samples were evaluated at 15 min, 60 min, and then every 60 min up to 12 h. If a requirement persisted, the samples were rechecked daily at 24 h, 48 h, and 72 h, then weekly for 21 d.

### 2.5. Heavy Metal Analysis

The design for this procedure was done according to the modification of a previously published study [21]. Heavy metal elements (Cr, Ni, Co, Cd, As, Hg, Pb, and Be) were considered for analysis in specimens since all these elements are shown to have some levels of carcinogenic potential. After evaluation of setting time analysis, the tooth samples were longitudinally sectioned and the set sealers from each tooth were collected on a glass plate. All the samples were prepared similarly and subjected to elemental analysis using inductively coupled plasma mass spectrometry (ICP-MS) (Agilent 7700x, Santa Clara, CA, USA) (Supplementary document Figure S3).The samples were repeated three times, and mean scores were calculated. For the analysis of the samples, they were exposed to microwave digestion for 45 min. In order to reduce the temperature and pressure inside the digestion vessel, a cooling phase was included at the end of the program. Followed by digestion, the solution appeared limpid without any suspended particles, with its volume seen to be the same before the digestion. After the digestion procedure, the mixture was subjected to 25 mL dilution with deionized and water and filtered. The final sample was subjected to ICP-MS analysis.

### 2.6. Statistical Analysis

The data were analyzed using IBM SPSS Statistics for Windows (version 23.0, IBM Corp. Armonk, NY, USA). The setting time evaluation was calculated for normality distribution using the Kolmogorov–Smirnov test and Shapiro–Wilk test for assessment and showed a parametric distribution. Descriptive statistics were done by converting all the values to mean and standard deviation. For the multivariate analysis, one way ANOVA with Tukey’s post hoc test was used for repeated measures. The repeated measures of ANOVA were used with Bonferroni correction to control the type 1 error during multiple comparisons. A *p*-value of less than 0.05 was considered a significant level.

## 3. Results

From the eight selected heavy metals classified as carcinogenic potential analyzed in this study, seven heavy metals were present in all test samples (Table 2). Cr at 5.24 ppm and as at 2.15 ppm was seen to be present at maximum levels in AHPlus sealer. This was seen to be reduced post exposure to 2% CHX (Cr at 0.84 ppm, As at 0.33 ppm), 17% EDTA (Cr at 0.50 ppm, As at 0.98 ppm), and 3% NaOCl (Cr at 0.38 ppm, As at 0.98 ppm). Overall, MTAFillapex and Tubli-Seal had the least number of heavy metals and did not significantly change post exposure to different irrigants. Although Sealapex had higher Hg (3.14 ppm) and Pb (8.66 ppm), Hg was not seen to be influenced post exposure to endodontic irrigants. On the other hand, Pb content drastically increased post exposure to 3% NaOCl (151.78 ppm) and 17% EDTA (346.83 ppm).

**Figure 2:**
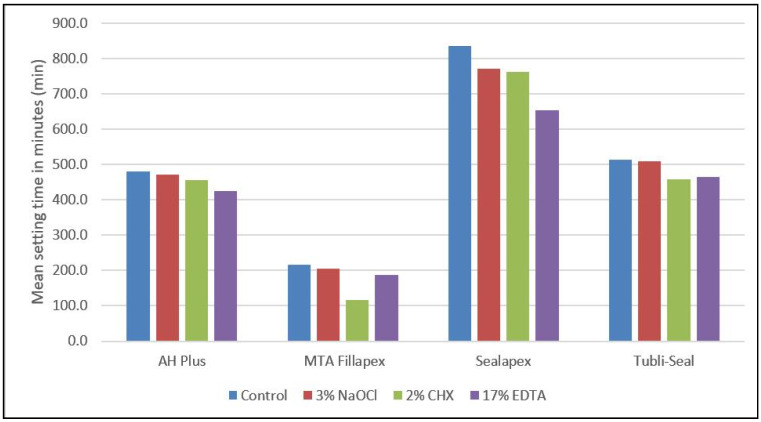
Bar chart denoting the mean setting time of different base endodontic sealers (AH Plus, MTA Fillapex, Sealapex, Tubli-Seal) on interaction with different endodontic irrigants (3% NaOCl, 2% CHX, and 17% EDTA) and distilled water.

Setting time of all sealers was different based on the different basesealers used (Table 3, Figure 2). On interaction with 3% NaOCl, all the sealers showed reduction in setting time (Table 3, Figure 2). On interaction with 17 % EDTA, it was seen that setting time was influenced at similar levels (Table 3, Figure 2). Overall reduction in setting time was more on interaction with 17% EDTA and 2% CHX, respectively. Intragroup comparison using a post hoc test showed statistical significance with different endodontic sealers on interaction with the selected endodontic irrigants (*p* < 0.05) (Table 4).

## 4. Discussion

The conjugated use of endodontic irrigants and sealers play a critical role in the success of endodontic treatment. The present study is a preliminary evaluation aiming to address if endodontic irrigants could influence the setting time and heavy metal composition of different base endodontic sealers on interaction. The null hypothesis proposed for this study was rejected since endodontic irrigants did influence the setting time and heavy metal composition of different tested endodontic sealers. To our knowledge, no prior reports have established if endodontic irrigants can influence the setting time and heavy metal characteristics of endodontic sealers on interaction. Setting time is considered to be a crucial property for endodontic sealers, with significant clinical implications since it influences the working time, flow rate, and cytotoxic and genotoxic properties of the sealer and can exhibit different values before the initial setting reaction [22]. Furthermore, variation in setting time can affect the sealers dentinal penetrability and specific properties, ultimately influencing the treatment outcome [23]. Prior studies have reported to some extent about influence of endodontic irrigants on the physicochemical properties of endodontic sealers [24], for instance, Rocha et al. [25] showed that irrigants such as 2% CHX and 2.5% NaOCl had influenced the properties of epoxy-based endodontic sealers’ bond strength on radicular dentin. Another study by de Assis et al. [26] reported that endodontic irrigants influenced sealers flowability and wettability properties on interaction with dentin. Though the studies, in general, reported on the influence of endodontic irrigants on sealer properties and their interaction with dentin, none discussed the chemical composition of sealer and its potential influence if variations were seen on the dentin adhesion mechanism.

This study was conducted on a tooth model with different final irrigation protocols since we wanted to closely replicate a clinical scenario where a clinician fails to follow a standard irrigation regimen, and to replicate the possible outcome when endodontic irrigants interacted with different base endodontic sealers. All the test samples were conducted on mandibular premolars since canal variations are seen to be significantly less with a single canal and a single portal of exit reported most of the time [27]. The selected teeth were decoronated, and curvatures less than 5° were selected since it would allow for effective penetration of endodontic irrigants till the predetermined working length and would not act as a confounding variable for the results of this study [28,29]. Based on multiple studies in different populations, it was witnessed that mandibular premolars commonly exhibit Vertucci’s type 1 configuration with a range of 70–85% [30,31,32] and mostly show an overall round canal shape, allowing for effective instrumentation, better irrigant penetration on the dentinal walls, and the removal of inorganic and organic components of the smear layer [33].

The composition and the variation of heavy metals for the different tested endodontic sealers was evaluated using ICP-MS technique. In this study, the variation in heavy metal levels was seen to be higher for sealers exposed to 3% NaOCl and 2% CHX than 17% EDTA. High heavy metal composition can have an untoward reaction to cells, further influencing the sealers biocompatibility factors and ultimately going against the notion of the sealer properties procured by the manufacturers. In the past, it has been reported that cells, on exposure to different base endodontic sealers, can exhibit diverse outcomes. For instance, it is seen that the different base endodontic sealers can have a direct influence on the pain levels of individuals since they depend on the individual cells’ ability to release inflammatory mediators [34]. In addition, sealers such as AH Plus containing epoxy resin and amines have organic compounds such as bisphenol diglycidyl ether that can have mutagenic potential with regard to cells [35]. All these factors that have been discussed can be explained to a certain extent due to the presence of heavy metals in these substances and their interactions with host cell.

Quantity of heavy metals and their increase in endodontic biomaterials could potentially influence the treatment outcome in the long run. This is due to the diverse nature of materials used for treatment procedures; all materials used could exhibit some heavy metals in their components due to the various ingredients used for the formulation process and have the ability to directly influence the human cells [36,37]. Though clinically these factors have not been reported, further research is necessary to establish a relationship concerning these outcomes.

According to the World Health Organization (WHO) classification, heavy metals such as Cr, Ni, Co, Cd, As, Hg, Pb, and Be are shown to be exhibit carcinogenic potential when in direct contact with human cells [38] and are the main factor for analysis in our study. Dentin discolouration is an unexpected outcome that can occur due to the prolonged interaction time of endodontic sealers [39] and is known to occur due to the variation and amount of heavy metal composition. Prior studies have signified the importance of heavy metals to an extent, for instance, Seok-Woo Chang et al. [16] reported that the presence of heavy metals in MTA-based materials was seen to be safe according to ISO 9917-1 standard. On the other hand, Kee-Yeon Kum et al. [40] reported varyingheavy metal compositions in various endodontic materials. In both studies, ICP-MS was the evaluation technique for heavy metal analysis, and its usage has been extended for numerous clinical and laboratory-based studies for the quantitative and qualitative determination of the number of heavy metal compounds present [41,42,43].

Different base endodontic sealers such as Tubli-Seal, AH Plus, Sealapex, and MTA Fillapex were used since they represent each category of different base endodontic sealer available currently in the market. It was seen that Sealapex showed the highest average setting time (834.6 min), followed by Tubli-Seal (514.7 min), AH Plus (479.6 min), and MTA Fillapex (215.7 min), respectively. The achieved results are in agreement with previously published data that showed high setting time for Sealapex [3]. Contrarily, on interaction with different endodontic irrigants, variation in setting times was seen. A drastic decrease in setting time was witnessed when AH Plus and Sealapex interacted with 17% EDTA; however, MTA Fillapex and Tubli-Seal did not exhibit similar interaction results with 17% EDTA but were seen on interaction with 2% CHX. This could be due to different amounts of accelerators and retarders present in the sealers that could have reacted with the irrigants during the setting phase [44,45].

The selected heavy metals for analysis were witnessed in varied levels and increased or decreased based on exposure to different endodontic irrigants. According to ISO 9917-1 standard used by manufacturers for the production of endodontic materials, regulation of the levels of As and Pb have already been placed; unfortunately, no such regulations were established for other heavy metals discussed prior and this is a matter of significant concern since reports have established that they have similar potential to cause carcinogenic and mutagenic changes to cells in the long run. In this study, Pb content was seen to be at high levels in general with Sealapex (8.66 ppm). On interaction with various irrigants—2% CHX (5.17 ppm), 3% NaOCl (151.78 ppm), and 17% EDTA (346.83 ppm)—the results were seen to diversify, possibly indicating that the properties of the sealers changed on interaction with endodontic irrigants. None of the tested sealers showed any Be amounts (0 ppm), and even on interaction with the selected irrigants, no variation in levels was witnessed. Cr, Ni, Co, Cd, As, Hg, and Pb were present in all the sealers in small quantities regardless of exposure to any irrigants. One of the significant findings in the present study was that AH Plus did not show any Hg (0.00 ppm) on interaction with endodontic irrigants; in 2% CHX (0.93 ppm), 3% NaOCl (1.54 ppm), and 17% EDTA (1.96 ppm), this element was seen to be present. Elemental mercury has been used for a considerable period of time for conservative procedures in the form of amalgam restoration, though recent evidence suggests that there is no correlation of toxicity issues such as nephrotoxicity to occur among individuals [46]. These results may not follow pertaining to endodontics since endodontic sealers have direct contact with periapical tissues and host cells and can have a have a direct effect on the cells; this is a topic for future research.

Similar to AH Plus, Sealapex also showed presence of As after interaction with 2% CHX and 3% NaOCl. Previously, As was used in endodontics for devitalizing inflamed pulp tissues; in recent times, its use has been ceased, with reports establishing potential toxic effects and complications to vital tissues [47]. It is imperative that clinicians must take precautions during the endodontic treatment procedure to avoid unprecedented events such as sealer extrusion, which potentially leads to cytotoxic and mutagenic changes to cells in the long run [48,49].Cr, on the other hand, is considered very toxic to human cells because it causes oxidative stress and increases the production of reactive oxygen species (ROS), leading to genomic DNA damage and deterioration of lipids and proteins [50,51]. All tested materials exhibited Cr at some levels and were seen to be highest in AH Plus (5.24 ppm). On exposure to different irrigants, there were variations in Cr levels in the test materials, showing that endodontic irrigants such as 2% CHX (0.84 ppm), 3% NaOCl (0.38 ppm), and 17% EDTA (0.50 ppm) could influence the level of heavy metals.Though the results were varied based on the interaction with various endodontic irrigants, it was witnessed overall that 17% EDTA, on interaction with AH Plus, Tubli-Seal, and MTA Fillapex, resulted in the fewest heavy metal element variations compared to other tested endodontic irrigants. This was not seen to be applicable with Sealapex since endodontic irrigants can significantly increase the quantity of heavy metals present.

## 5. Strength, Limitations, and Future Directions

The strength of this study is that it can be described as a first of its kind in endodontic literature since it documents the possible influence of setting time and heavy metal differences with endodontic sealers on interaction with endodontic irrigants. The levels of metal composition varied with different materials on interaction. Metal compounds, though, are present in all endodontic sealers, and their values can vary on interaction with different irrigants used in endodontics, eventually influencing the outcome in the long run. One of the limitations of the present study is the use of a tooth model; though it better simulates clinical conditions, the results achieved could vary when done in vivo, based on a multitude of factors such as clinician’s irrigation preferences, treatment protocol, endodontic irrigant concentration, the tooth of treatment, and canal morphology [52,53]. Though the use of different final irrigation protocols in the assessed teeth would not necessarily replicate the clinical scenario, it was nevertheless conducted since the primary aim was to assess if endodontic irrigants present on the internal dentinal surface had an influence on the properties of the endodontic sealers. This also could be considered as a limitation to a certain extent since not all practitioners would follow the similar irrigation regimens shown in this study and can vary with different practitioners. Another factor was the limitation of only analyzing the initial set of different base endodontic sealers since the study was designed to assess the setting times of different sealers on interaction with endodontic irrigants; we achieved significant differences on assessing the initial set of the sealer and did require further assessment of final setting time. Another limitation is the sealers used; the results emulated may not necessarily be replicated when used with other commercially available different base endodontic sealers. Being a preliminary based in vitro investigation, further research is needed in this respect to establish if these factors are seen with other commercially available sealers in other clinical scenarios.

## 6. Conclusions

Within the limitations of this study, the setting times of MTA Fillapex, AH Plus, Tubli-Seal, and Sealapex are seen to be significantly influenced on interaction with selected endodontic irrigants most commonly used in clinical practice. The preliminary results show that heavy metals were present in all the tested sealers and that the levels were greatly influenced by interaction with endodontic irrigants. In a clinical scenario, the clinician must take the utmost care to conduct a final irrigation with saline and dry the canal completely to avoid any traces of endodontic irrigant before obturation, potentially leading to cytotoxic and toxic effects on periapical cells in the long run. Further analysis is needed to determine the results achieved from the present study, which can differ based on different clinical scenarios.

## Figures and Tables

**Table 1:** Root canal sealers used in the present study.

Materials	Composition	Manufacturer
Sealapex	calcium hydroxide, barium sulfate, zinc oxide, titanium dioxide, and zinc stearate.	SybronEndo, Orange, CA, USA
AH Plus	Paste A:bisphenol A epoxy resin, bisphenol F epoxy resin, calcium tungstate, zirconium oxide, aerosol, and iron oxide Paste B: dibenzyldiamine, adamantane amine, tricyclodecane-diamine, calcium tungstate, zirconium oxide, aerosol, and silicon oil	Dentsply DeTrey GmbH, Konstanz, Germany
MTA Fillapex	After mixing: salicylate resin, natural resin, diluting resin, bismuth trioxide, nanoparticulated silica, MTA, and pigments.	Angelus Soluções Odontológicas, Londrina, Brazil
Tubli-Seal	Base:zinc oxide, oleo resin, bismuth trioxide, thymol iodide, oils, and waxes. Catalyst:eugenol, polymerized resin, and annidalin	Kerr Dental, Orange, CA, USA

**Table 2:** Heavy elements present in different base endodontic sealers after assessment with inductively coupled plasma mass spectrometry (ICP-MS).

Elements (ppm)(Mean ± SD)	AH Plus	AH Plus	AH Plus	AH Plus	MTA Fillapex	MTA Fillapex	MTA Fillapex	MTA Fillapex	Tubli-Seal	Tubli-Seal	Tubli-Seal	Tubli-Seal	Sealapex	Sealapex	Sealapex	Sealapex
	-	CHX	NaOCl	EDTA	-	CHX	NaOCl	EDTA	-	CHX	NaOCl	EDTA	-	CHX	NaOCl	EDTA
**Cr**	5.24 ± 1.24	0.84 ± 0.005	0.38 ± 0.006	0.50 ± 0.002	1.35 ± 0.08	0.31 ± 0.0002	0.71 ± 0.0002	0.71 ± 0.0002	1.42 ± 0.05	0.79 ± 0.08	0.96 ± 0.006	1.42 ± 0.85	0.22 ± 0.007	0.44 ± 0.04	0.93 ± 0.0006	0.78 ± 0.008
**Ni**	0.51 ± 0.01	0.30 ± 0.005	0.91 ± 0.009	0.38 ± 0.069	2.84 ± 1.08	0.23 ± 0.45	1.45 ± 0.81	0.36 ± 0.08	0.56 ± 0.08	0.10 ± 0.0002	1.54 ± 0.08	1.47 ± 0.65	0.17 ± 0.005	0.17 ± 0.02	0.66 ± 0.001	1.35 ±0.18
**Co**	0.08 ± 0.005	0.09 ± 0.002	0.18 ± 0.001	0.07 ± 0.0002	0.58 ± 0.006	0.05 ± 0.00004	0.26 ± 0.10	0.13 ± 0.002	0.13 ± 0.001	0.04 ± 0.0002	0.31 ± 0.001	0.28 ± 0.002	0.04 ± 0.0001	0.04 ± 0.0001	0.09 ±0.0005	0.24 ± 0.10
**Cd**	0.08 ± 0.005	0.06 ± 0.002	0.05 ± 0.0004	0.17 ± 0.0012	0.29 ± 0.08	0.08 ± 0.0002	0.08 ± 0.0002	0.07 ± 0.002	0.13 ± 0.045	0.09 ± 0.001	0.13 ± 0.0221	0.09 ± 0.0002	0.09 ± 0.0002	0.04 ± 0.0001	0.09 ± 0.0005	0.10 ± 0.002
**As**	2.15 ± 0.55	0.33 ± 0.15	0.29 ± 0.0085	0.98 ± 0.034	2.26 ± 1.00002	0.45 ± 0.0002	1.01 ± 0.45	0.51 ± 0.006	2.02 ± 0.18	0.81 ± 0.002	1.20 ± 0.08	1.62 ± 0.15	0.00	0.17 ± 0.0023	0.45 ± 0.002	0.00
**Hg**	0.00	0.93 ± 0.45	1.54 ± 0.65	1.96 ± 0.04	2.79 ± 0.02	1.19 ± 0.006	1.98 ± 0.91	1.07 ± 0.058	1.55 ± 0.045	1.07 ± 0.004	1.75 ± 0.78	1.14 ± 0.28	3.14 ± 1.02	3.36 ± 0.006	0.27 ± 0.002	1.49 ± 0.59
**Pb**	0.34 ± 0.05	0.18 ± 0.08	0.22 ± 0.04	0.19 ± 0.002	1.01 ± 0.02	0.09 ± 0.05	0.4 ± 0.002	0.23 ± 0. 005	0.43 ± 0.004	0.16 ± 0.002	0.70 ± 0.25	0.50 ± 0.20	8.66 ± 3.02	5.17 ± 2.05	151.78 ± 22.45	346.83 ± 28.65
**Be**	0.00	0.00	0.00	0.00	0.00	0.00	0.00	0.00	0.00	0.00	0.00	0.00	0.00	0.00	0.00	0.00

**Table 3:** Mean and standard deviation of the setting time of different endodontic base sealers and on interaction with commonly used endodontic irrigants in minutes (min). One way ANOVA test showed a statistically significant difference in all groups (*p* < 0.05).

Endodontic Irrigants	Endodontic Sealers	Mean	Standard Deviation	F-Value	*p*-Value
No interaction with any endodontic irrigants	AH Plus	479.6	10.2	834.50	0.0005 **
	MTA Fillapex	215.7	6.3		
	Sealapex	834.6	26.8		
	Tubli-Seal	514.7	7.9		
On interaction with 3% NaOCl	AH Plus	471.5	17.4	239.65	0.0005 **
	MTA Fillapex	204.9	4.2		
	Sealapex	772.0	48.6		
	Tubli-Seal	509.9	3.7		
On interaction with 2% CHX	AH Plus	456.4	11.7	311.06	0.0005 **
	MTA Fillapex	115.7	6.2		
	Sealapex	762.1	49.9		
	Tubli-Seal	457.8	4.6		
On interaction with 17% EDTA	AH Plus	423.9	11.3	816.45	0.0005 **
	MTA Fillapex	187.3	4.5		
	Sealapex	652.6	19.4		
	Tubli-Seal	465.2	4.0		

** Statistically Significant (*p* < 0.01).

**Table 4:** Intragroup comparison using post hoc tukey test showed a significant difference in all the test groups (*p* < 0.05) except for AH Plus-Tubli-Seal group (*p* > 0.05) on interaction with 3% NaOCl and 2% CHX.

Dependent Variable			MD (I-J)	Std. Error	*p*-Value	95% C.I	
						LB	UB
Control	AH Plus	MTA Fillapex	263.9000	12.414	0.0005 **	224.1	303.7
		Sealapex	−355.0000	12.414	0.0005 **	−394.8	−315.2
		Tubli-Seal	−35.1000	12.414	0.085 ^#^	−74.9	4.7
	MTA Fillapex	Sealapex	−618.9000	12.414	0.0005 **	−658.7	−579.1
		Tubli-Seal	−299.0000	12.414	0.0005 **	−338.8	−259.2
	Sealapex	Tubli-Seal	319.9000	12.414	0.0005 **	280.1	359.7
3% NaOCl	AH Plus	MTA Fillapex	266.6000	21.20	0.0005 **	198.7	334.5
		Sealapex	−300.5000	21.20	0.0005 **	−368.4	−232.6
		Tubli-Seal	−38.4000	21.20	0.335 ^#^	−106.3	29.5
	MTA Fillapex	Sealapex	−567.1000	21.20	0.0005 **	−635.0	−499.2
		Tubli-Seal	−305.0000	21.20	0.0005 **	−372.9	−237.1
	Sealapex	Tubli-Seal	262.1000	21.20	0.0005 **	194.2	330.0
2% CHX	AH Plus	MTA Fillapex	340.7000	21.18	0.0005 **	272.9	408.5
		Sealapex	−305.7333	21.18	0.0005 **	−373.6	−237.9
		Tubli-Seal	−1.4000	21.18	1.00 ^#^	−69.2	66.4
	MTA Fillapex	Sealapex	−646.4333	21.18	0.0005 **	−714.3	−578.6
		Tubli-Seal	−342.1000	21.18	0.0005 **	−409.9	−274.3
	Sealapex	Tubli-Seal	304.3333	21.18	0.0005 **	236.5	372.2
17% EDTA	AH Plus	MTA Fillapex	236.6000	9.47	0.0005 **	206.29	266.91
		Sealapex	−228.7333	9.47	0.0005 **	−259.05	−198.42
		Tubli-Seal	−41.3000	9.47	0.010 **	−71.61	−10.99
	MTA Fillapex	Sealapex	−465.3333	9.47	0.0005 **	−495.65	−435.02
		Tubli-Seal	−277.9000	9.47	0.0005 **	−308.21	−247.59
	Sealapex	Tubli-Seal	187.4333	9.47	0.0005 **	157.12	217.75

** Statistically Significant (*p* < 0.01) ^#^ no statistical significance (*p* > 0.05).

## Data Availability

The data set used in the current study will be made available on reasonable request.

## Supplementary Materials

The following are available online at https://www.mdpi.com/article/10.3390/polym14010027/s1, Figure S1: Mandibular premolar specimens mounted on wax molds followed by instrumentation using Protaper Gold rotary system (Dentsply Maillefer, Ballaigues, Switzerland) (1A) and filling the canal with respective sealers using a motorized lentulospiral (Dentsply Maillefer, Ballaigues, Switzerland) (1B); Figure S2: Cross section of the decoronated tooth with sealer filled till the orifice using a size 25 motorized lentulospiral (Dentsply Maillefer, Ballaigues, Switzerland); Figure S3: Analysis of heavy metal elements in the sealer conducted using inductively coupled plasma mass spectrometry (ICP-MS) (Agilent 7700x, Santa Clara, CA, USA).
