# Supplementary material for: Influence of Commonly Used Endodontic Irrigants on the Setting Time and Metal Composition of Various Base Endodontic Sealers
Source: Polymers (Basel). 2021 Dec 22;14(1):27. doi: 10.3390/polym14010027 (PMC8747268; doi:10.3390/polym14010027)

Supplementary documents

Figure S1: Mandibular premolar specimens mounted on wax molds followed by instrumentation using Protaper Gold rotary system (Dentsply Maillefer, Ballaigues, Switzerland) (1A) and filling the canal with respective sealers using a motorized lentulospiral (Dentsply Maillefer, Ballaigues, Switzerland) (1B).

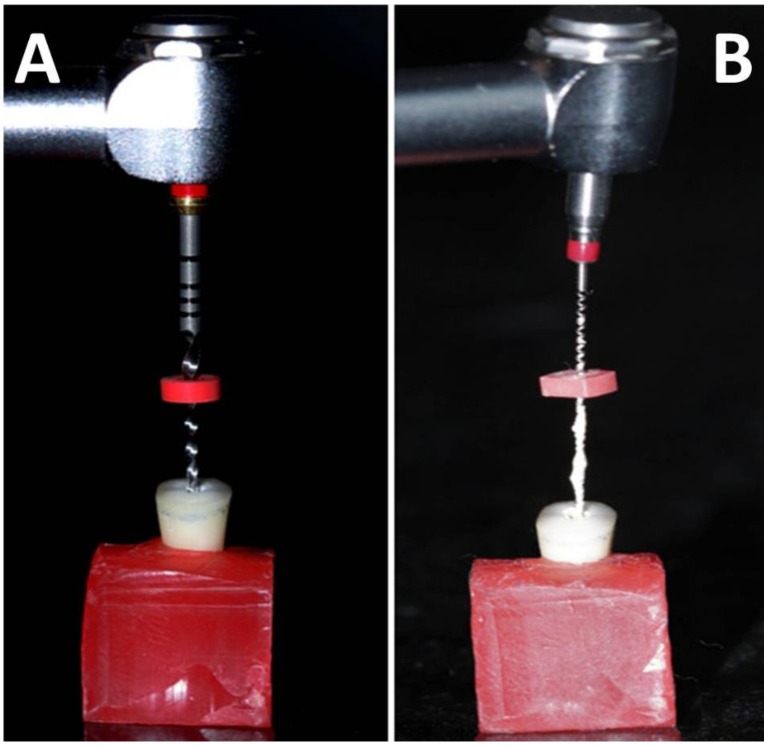

Figure S2: Cross section of the decoronated tooth with sealer filled till the orifice using a size 25 motorized lentulospiral (Dentsply Maillefer, Ballaigues, Switzerland)

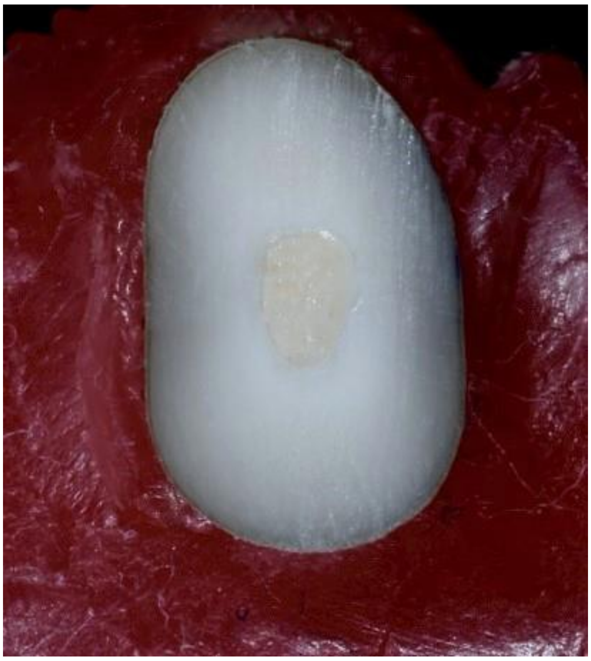

Figure S3: Analysis of heavy metal elements in the sealer conducted using inductively coupled plasma mass spectrometry (ICP-MS) (Agilent 7700x, Santa Clara, CA, USA)

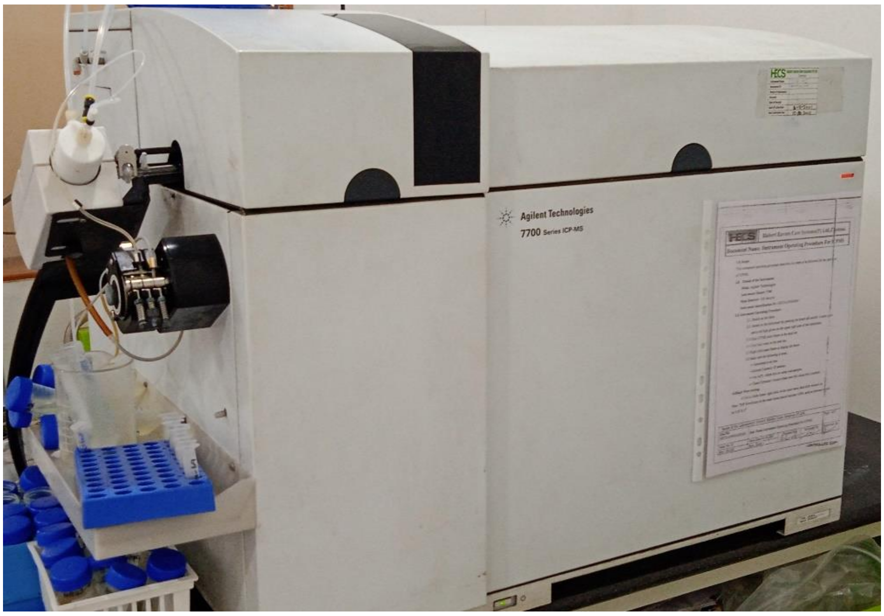

Supplement: Supplementary file 1 [file polymers-14-00027-s001.zip › polymers-1490990-supplementary.pdf]
